# Spiritual Care Therapy on Quality of Life in Cancer Patients and Their Caregivers: A Prospective Non-randomized Single-Cohort Study

**DOI:** 10.1007/s10943-016-0324-6

**Published:** 2016-11-16

**Authors:** A. Sankhe, K. Dalal, V. Agarwal, P. Sarve

**Affiliations:** Clinical Research, BhaktiVedanta Hospital and Research Institute, Srishti Complex, Mira Road, Thane, Mumbai, 401107 Maharashtra India

**Keywords:** Spiritual care, Palliative care, Oncology

## Abstract

Spiritual care is still in infancy stage all over the globe including India. The present study was an original study evaluating the role of spiritual care in cancer patients and their primary caregivers regarding their spiritual and general well-being. The study was a prospective, non-randomized single-group study involving cancer patients undergoing surgery and their primary caregivers. Functional assessment of cancer therapy—general and functional assessment of chronic illness therapy-spiritual care was evaluated during the admission and at the time of discharge, two, four  and 6 months following discharge from the hospital. Descriptive statistics was used for demographic details and repeated measure ANOVA with Dunn’s test was used for analysis of changes in the scores. A total of 107 (63 males and 44 females) patients with a mean (SD) of age 51 (13) years were recruited in the study. Similarly, for each patient one of their primary caregivers was recruited with their mean (SD) age of 39.4 (12.7) years. A total of 11/107 (10.3%) patients died and nine out of 107 (8.4%) were lost to follow-up eventually during the study period. There was a statistically significant (*P* < 0.0001) increase in the scores at all the follow-up periods in both the patient and their relative groups. To conclude, we found out that spiritual care on the basis of MATCH guideline improved the level of not only spiritual well-being but general well-being also in both the patients and their primary caregivers. Control group could have improved scientific validity of study in accessing effect of spiritual care. Authors believe that more robust comparative study on each principle against all five MATCH principles in future will add scientific validity and clear the various ambiguities in spiritual care.

## Introduction

Cancer is one of the serious critical health problems where despite many advances in the therapeutics, mortality is inevitable. A total of 14 million new cases and 8.2 million cancer-related deaths were reported worldwide in 2012 (World Cancer Report 2014). Both the infection-related and lifestyle-related cancers are increasing posing a major obstacle to human development and well-being. The battle against cancer is the most difficult task with hardly any cure and recently focus has shifted toward prevention strategies and palliative care.

Palliative care includes strategies toward relief of pain, symptoms and emotional stress. Anticipating the demands of sustaining a cancer-affected life and eventually possible end-of-life care can help ease the journey from care and grief toward acceptance and healing (Sherman 1998). In addition to the continuation of treatment of patients with therapeutic agents, the focus mainly has shifted in making the patient as comfortable as possible improving their quality of life and their caregivers. A cancer patient will experience the worst symptoms not only because of the disease condition but also due to the toxicity of anticancer drugs ultimately leaving a poor quality of life (Numbers and Amundsen 1986). The unique challenges associated with cancer treatment and end-of-life care include both the moral and financial support that is being provided from a variety of sources such as home health agents, nursing home personnel, hospice providers and palliative care physicians (Walter 2002). Both the patients and their primary caregivers often land up in having a significant emotional, social and spiritual distress. Of these, the spiritual distress in cancer patient may result from the belief that their ailment is a punishment from God and in few instances patients lose their faith in the practice of spirituality ultimately. To achieve the complete health as per World Health Organization definition, it is essential to have a spiritual and general well-being. With this background, the present study has been conducted to assess the effect of spiritual care on the domain of spiritual and general well-being in both the cancer patients receiving cancer treatment and their relatives.

## Methods

The study was conducted after obtaining approval from institutional ethics committee between October 2013 and September 2014. Written informed consent was obtained from the study participants as well as their caregivers. Adult patients aged between 18 and 65 years of either sex and diagnosed as having cancer undergoing surgery were included. Patients with an expected life expectancy of less than 6 months were excluded from the study. BhaktiVedanta Hospital is a private healthcare center catering all kind of patients attending the hospital. A dedicated team including physicians, staff nurses and a spiritual care specialist are involved in delivering spiritual care to patients. Spiritual care was given under MATCH guidelines. MATCH guidelines as defined below is being used to deliver spiritual care.

### MATCH Guidelines

- *M—Mercy* Mercy principle does not promote violent slaughter of innocent animals but entails our existence in this universe with minimal violence to other living beings and environment We have counseled subjects and their relatives to follow vegetarian diet and refrain from non-vegetarian diet as non-vegetarian diet has more carcinogens and proved to be associated with many cancers. Also vegetarian diet has more antioxidants which will help the cellular tissues
- *A—Austerity* We counseled the subjects and relatives to accept the reality of the disease and have a positive mindset through spiritual practices instead of taking temporary shelter of intoxication such as smoking, tobacco chewing and alcoholism and asked them to avoid
- *T—Truthfulness* We counseled subjects and their relatives to have straight forward dealings with everyone and avoid stress
- *C—Cleanliness* We have counseled subjects and relatives to maintain good hygiene to avoid infections
- *H—Holy name* We counseled the patients and relatives to pray and meditate on the Holy names of God (keeping their faith in their religion)

The principles of the BhaktiVedanta Hospital model for spiritual care are based on the following:

1. No discrimination on the basis of religion, sex, age or belief in God
2. Accepting the common broad principles of all major religions
3. Care is manifested through environment such as vegetarian, wholesome food, spiritual sound vibration and emotional care.

The spiritual care for all the study participants were administered for a period of one and half hour daily (counseling, reading and chanting for 30 min each). We used two scales for measurement of quality of life of patients and their caregivers. Both the tools are protected by copyrights, and permission from the original authors was obtained for using the same in the present study. One was Functional Assessment of Cancer Therapy—General (FACT-G), a 27-item compilation of questions divided into four primary quality of life domains: physical well-being, social/family well-being, emotional well-being and functional well-being. It is a well-validated scale found to be appropriate for use in patients with any form of cancer. Another validated scale used in the study was Functional Assessment of Chronic Illness Therapy—spiritual well-being (FACIT-Sp) (Webster et al. 2003). The scores were evaluated at the baseline, discharge and two, four  and 6 months following the surgery. Descriptive statistics was used for representing the demographic data, and repeated measures ANOVA test was used for analyzing the difference of scores at various time points.

## Results

### Demographic Details

A total of 107 (63 males and 44 females) patients with a mean (SD) of age 51 (13) years were recruited in the study. Similarly, for each patient one of their primary caregivers was recruited with their mean (SD) age of 39.4 (12.7) years. A total of 11/107 (10.3%) patients died and nine out of 107 (8.4%) were lost to follow-up eventually during the study period. As regards to the diagnoses of the study patients, carcinoma breast was observed in 24, carcinoma buccal mucosa in 50, carcinoma tongue in eight, carcinoma ovary in five, carcinoma prostate in three, squamous cell carcinoma in two, carcinoma mandible in two and renal cell carcinoma in two, and one each had appendicular, pancreatic, colon, scalp, esophagus, RMT, stomach, rectum, cervix and leukoplakia.

### Change in Scores

Both FACT-G and FACIT-Sp scores were significantly different in both the patient and their caregivers group at all the follow-up time points as compared to baseline. Table 1 lists the score values at different time points in both the groups of study participants. Additionally, in FACT-G, significant improvements were observed in all the domains from baseline both in the patients and their caregivers (Table 2). One of the glaring findings was that one of the subjects became apprehensive after revealing cancer diagnosis to caregiver; however, subject has accepted reality after MATCH guideline spiritual counseling.

**Table 1:** FACT-G and FACIT-Sp scores between the study participants

Visit time points	Admission	Discharge	Follow-up 1 (2 months)	Follow-up 2 (4 months)	Follow-up 3 (6 months)
*FACT-G in patients*					
Mean (SD)	59.9 (26)	88.1 (17)	88.1 (19.2)	92.9 (19.5)	98.1 (15.8)
*FACIT-Sp in patients*					
Mean (SD)	28.5 (13.3)	42.5 (5.6)	42.6 (6.6)	43.5 (6.8)	45.4 (5)
*FACT-G in patients’ caregiver*					
Mean (SD)	60.3 (24.7)	90.2 (20.2)	95.6 (15.9)	97.7 (14.4)	98.1 (16.7)
*FACIT-Sp in patients’ caregivers*					
Mean (SD)	29.7 (11.4)	41.2 (8.4)	43.2 (6.9)	44.5 (5.7)	45.4 (4.8)

Statistical analysis: repeated measures ANOVA with post hoc Dunn’s test—*P* < 0.0001

**Table 2:** Analysis of domains in FACT-G scores between the study participants [values are mentioned in mean (SD)]

Type of domains	Admission	Discharge	Follow-up 1 (2 months)	Follow-up 2 (4 months)	Follow-up 3 (6 months)	*P* value
*Patients*						
Physical well-being	15 (8.1)	21.5 (5.8)	21.9 (6.2)	23.4 (6.3)	24.9 (5.1)	<0.0001
Social well-being	18.1 (6.4)	24.1 (4.8)	25 (4.6)	25.7 (3.5)	26.6 (2.4)	<0.0001
Emotional well-being	11.4 (7.6)	20.6 (4)	20.5 (4.7)	21 (5.4)	22.2 (4.7)	<0.0001
Functional well-being	14.2 (7.3)	20.9 (6.7)	20.9 (7.3)	24.4 (22.1)	24.8 (5.3)	<0.0001
*Patients’ caregiver*						
Physical well-being	16 (8.1)	24.4 (4.9)	24.7 (4.9)	25.9 (3.4)	26.3 (3.1)	<0.0001
Social well-being	18.4 (6.6)	24.3 (5.1)	25.4 (4.1)	26.1 (3)	26.6 (3)	<0.0001
Emotional well-being	10.7 (7.3)	21 (4)	21.2 (3.8)	21.5 (5.1)	21.7 (5.4)	<0.0001
Functional well-being	16 (6.8)	25.5 (19.7)	24.2 (5.8)	25.3 (5.1)	25.8 (4.8)	<0.0001

## Discussion

The present study was performed to evaluate the effect of spiritual care on the spiritual and general well-being of cancer patients and their primary caregivers. We found out that there is a significant increase in the scores at all the follow-up times when compared to the baseline values indicating a significant improvement in the well-being in both the groups of study participants.

Both the patients receiving palliative care and their families are profoundly affected by the challenges of the illness and the adverse effects encountered during the treatment of the same. As the family members also observe the kind of treatment/care provided in the hospital set up, they get exposed to the type of support/assistance offered and in today’s world where the decision making of treating any illness is based on sharing between both the healthcare personnel and patient, it is logical for them to get distressed. Also, patients with incurable illness suffer not only physical problems but also have psychological stress. Santo et al. (2011) did an assessment of amount of burden and the quality of life of caregivers of children with cancers and found that nearly 60% of them had significant affect on emotion, vitality and suffered pain. Kim and Given (2008) did a systematic review of different studies evaluating the literature published between 1996 and 2007 reiterated the fact that not only the patients but their caregivers also were affected by the burden of cancer. But another systematic review of knowledge of caregivers related to the care for patients with terminal illness from a Southeast Asian country revealed a poor knowledge necessitating a need for constant and regular counseling. Meecharoen et al. (2013) Additionally, Yang et al. (2012) evaluated the factors associated with quality of life among the caregivers and found that mental quality of health to be seriously impaired than physical quality of life. Not only the quality of life is affected by disease condition but also chemotherapy induces adverse effects affecting the same. Vrettos et al. (2012) also found out that greater proportion (80%) of patients had their relatives with either moderate or extreme anxiety or depression. Surprisingly, the amount of stress level among the caregiver is not different between various stages of cancer (Grov and Valeberg 2012). Not only the physical stress of patient troubles their caregivers but also there may be economic distress due to the cost of chemotherapy. A recent estimate suggests that Canada’s 1.5–2 million family caregivers provide $25–$26 billion worth of care annually and incur $80 million in out-of-pocket expenses annually (Hollander et al. 2009). It is crucial to address their quality of life, physical and psychological well-being the alter being the core of spiritual care (El-Jawahri et al. 2011). Unfortunately, even from developed nations it has been estimated that only around 67% of patients in their end-of-life care die without having any access to spiritual care (Teno et al. 2004). Also, still ambiguities remain on who should be the provider of spiritual care as few studies have evaluated the care administered by physicians (Lo et al. 2002) and few others with nurses (Fehring et al. 1997). Any spiritual care should explicitly be away from religious beliefs. Understanding the importance, the Institute Of Medicine, WHO and National Hospice and Palliative Care Organization recommend spiritual care as a palliative measure (Field and Cassel 1997; World Health Organization 2015) Lack of time was reported as the major barrier for implementation of spiritual care (Daaleman et al. 2008). Very few institutions around the world have facilities to impart spiritual care complying the need of hour in the healthcare arena.

Authors believe that spiritual care on the basis of MATCH guideline has helped subjects and their relative for improved quality of life and spiritual well-being. Spiritual care with principles of Mercy, Austerity, Truthfulness, Cleanliness and Holy Name has been portrayed to be one of the means of reducing or even eliminating such distress both in the patients and their primary caregivers. Austerity is one of the important principles of MATCH guideline where authors suggested that intoxication distracts subjects and enter into ‘World of dreams’ and ultimately is taking subjects away from reality of life which was supported in earlier study (Calhoun et al. 2005). In case of cancer, subjects are usually in mode of denial who need to accept reality and face ‘fear of death.’ The literature suggested fear of death can be countered by Mantra meditation chanting which leads to decrease stress and ‘fear of death’ (Shannahoff-Khalsa 2005; Cunningham 2005). This supported principle of Holy name chanting of MATCH guideline. Non-vegetarian diet was proved to be intrinsic factor in causing cancers such as gastric and colon. Vegetarian food has comparatively more antioxidants (Ames et al. 1995). Some cancers have genetic predisposition which may cascade to future generations (Futreal et al. 2004). Defining Mercy and Austerity principles, authors had taken opportunity to counsel caregivers emphasizing on vegetarian diet and refrain from intoxication. As per most of oncologist consensus, most of cancer patients are hesitant to accept reality or reveal truth to caregivers. One of the subject’s findings from our study about not revealing disease to caregiver had lead to distress; however, spiritual care counseling emphasizing principle of Truthfulness made subjects emotionally stable by accepting reality and revealing truth to caregivers. The literature has well accepted that cancer patients are immuno-compromised and more prone to infections (Bur et al. 2016). Authors had encouraged patients to maintain physical hygiene through principle of Cleanliness. There are many studies in the literature where each principle of MATCH guideline was assessed individually; however, this study had attempted to assess all five principles together under umbrella of MATCH guidelines (Calhoun et al. 2005; Shannahoff-Khalsa 2005; Cunningham 2005; Ames et al. 1995; Futreal et al. 2004; Bur et al. 2016).

To conclude, we found out that spiritual care on the basis of MATCH guidelines improved the level of not only spiritual well-being but general well-being also in both the patients and their primary caregivers. Control group could have improved scientific validity of study in accessing effect of spiritual care. Authors believe that more robust comparative study on each principle against all five MATCH principles in future will add scientific validity and clear the various ambiguities in spiritual care.
